# *N*-(2-Benzoyl-4-chloro­phen­yl)-4-chloro­benzene­sulfonamide

**DOI:** 10.1107/S1600536808007435

**Published:** 2008-03-29

**Authors:** Arto Valkonen, Ryszard Gawinecki, Henryk Janota, Borys Ośmiałowski, Erkki Kolehmainen

**Affiliations:** a Department of Chemistry, University of Jyväskylä, PO Box 35, FIN-40014 Jyväskylä, Finland; b Department of Chemistry, University of Technology and Life Sciences, Seminaryjna 3, PL-85-326 Bydgoszcz, Poland

## Abstract

The title compound, C_19_H_13_Cl_2_NO_3_S, is an *N*-aryl­sulfonyl derivative of 2-amino-5-chloro­benzophenone. The compound is biologically active and shows potential to be utilized as an inhibitor of CCR2 and CCR9 receptor functions. In the crystal structure, there is an intra­molecular N—H⋯O hydrogen bond between the amide and carbonyl groups. The benzoyl and 4-chloro­phenyl groups form intra­molecular and inter­molecular face-to-face contacts, with a dihedral angle of 10.6 (1)° between their mean planes in both cases, and centroid–centroid separations of 4.00 (1) and 4.25 (1) Å for the intra- and inter­molecular inter­actions, respectively.

## Related literature

For related literature, see: Basak *et al.* (2008 ▶); Fleming *et al.* (2003 ▶); Kolehmainen *et al.* (2003 ▶); Sternbach *et al.* (1962 ▶).
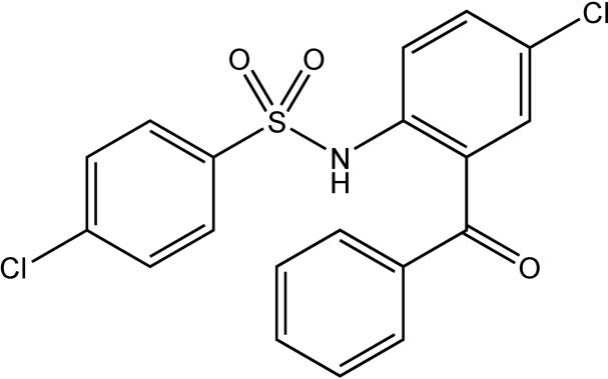


## Experimental

### 

#### Crystal data

C_19_H_13_Cl_2_NO_3_S
*M* *_r_* = 406.26
Monoclinic, 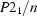

*a* = 8.2307 (1) Å
*b* = 18.5014 (3) Å
*c* = 12.1364 (2) Å
β = 105.211 (1)°
*V* = 1783.38 (5) Å^3^
*Z* = 4
Mo *K*α radiation
μ = 0.50 mm^−1^
*T* = 173 (2) K
0.25 × 0.25 × 0.15 mm

#### Data collection

Bruker Kappa APEXII CCD diffractometer
Absorption correction: none
13987 measured reflections
4401 independent reflections
3534 reflections with *I* > 2σ(*I*)
*R*_int_ = 0.044

#### Refinement

*R*[*F* ^2^ > 2σ(*F* ^2^)] = 0.042
*wR*(*F* ^2^) = 0.084
*S* = 1.05
4401 reflections
238 parameters
1 restraint
H atoms treated by a mixture of independent and constrained refinement
Δρ_max_ = 0.32 e Å^−3^
Δρ_min_ = −0.31 e Å^−3^

### 

Data collection: *COLLECT* (Bruker, 2004 ▶); cell refinement: *DENZO-SMN* (Otwinowski & Minor, 1997 ▶); data reduction: *DENZO-SMN*; program(s) used to solve structure: *SIR2002* (Burla *et al.*, 2003 ▶); program(s) used to refine structure: *SHELXL97* (Sheldrick, 2008 ▶); molecular graphics: *ORTEP-3 for Windows* (Farrugia, 1997 ▶); software used to prepare material for publication: *SHELXL97*, *PLATON* (Spek, 2003 ▶) and *Mercury* (Macrae *et al.*, 2006 ▶).

## Supplementary Material

Crystal structure: contains datablocks global, I. DOI: 10.1107/S1600536808007435/bi2286sup1.cif

Structure factors: contains datablocks I. DOI: 10.1107/S1600536808007435/bi2286Isup2.hkl

Additional supplementary materials:  crystallographic information; 3D view; checkCIF report

## Figures and Tables

**Table 1:** Hydrogen-bond geometry (Å, °)

*D*—H⋯*A*	*D*—H	H⋯*A*	*D*⋯*A*	*D*—H⋯*A*
N1—H1⋯O1	0.865 (15)	2.20 (2)	2.798 (2)	126.1 (18)

**Fig. 1.**
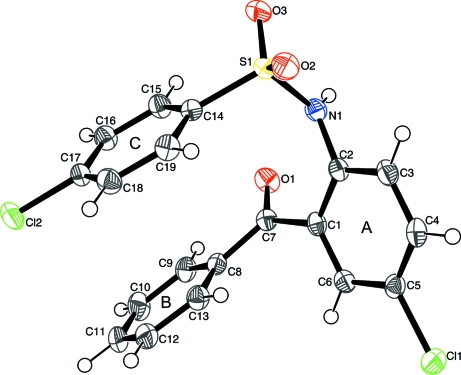
The molecular structure of the title compound with displacement ellipsoids drawn at the 50% probability level. H atoms are represented by circles of arbitrary size

**Fig. 2.**
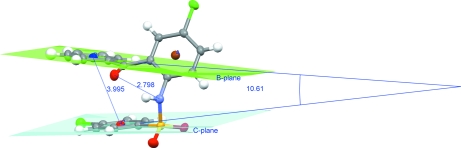
Molecular conformation with selected geometric parameters

**Fig. 3.**
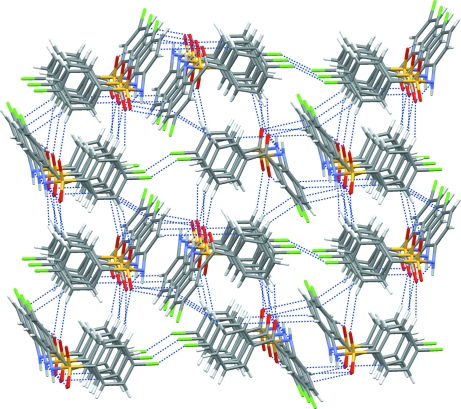
Packing viewed along the a-axis showing the stack-like architecture

## supplementary crystallographic information

### Comment

The title compound was originally prepared to study its molecular structure by spectroscopy (Kolehmainen *et al.*, 2003). The compound has a sulfone group showing strong electron acceptor capability and two S=O double bonds with ineffective conjugation properties with other double bonds. The compound has also shown a potential to be utilized as inhibitor of CCR2 (Basak *et al.*, 2008) and CCR9 (Fleming *et al.*, 2003) receptor functions. This potent antagonist can possibly be utilized in pharmaceutical compositions for treatment of CCR2 and CCR9 related diseases.

In the crystal, there is an intramolecular N—H···O hydrogen bond between the amide and carbonyl groups (Fig. 1). The benzoyl and 4-chlorophenyl groups form intramolecular (Fig. 2) and intermolecular face-to-face contacts (Fig. 3), with a dihedral angle of 10.6 (1)° between their mean planes in both cases, and a centroid-centroid separation of 4.00 (1) and 4.25 (1)Å for the intra- and intermolecular interactions, respectively.

### Experimental

The title compound was obtained by condensation of 2-amino-5-chlorobenzophenone and 4-chlorobenzenesulfonyl chloride according to a previously described method (Sternbach *et al.*, 1962). The reaction product was purified by crystallization from ethanol. The spectroscopic characterization (NMR, IR) has previously been reported by us (Kolehmainen *et al.*, 2003). The single-crystal suitable for X-ray determination was obtained by extremely slow evaporation of a CDCl_3_ solution in a NMR tube.

### Refinement

All H atoms were visible in electron density maps, but those bound to C were placed in idealized positions and allowed to ride on their parent atoms at C—H distances of 0.95 Å with *U*_iso_(H) = 1.2*U*_eq_(C). The position of the N—H proton was refined with the N—H distance restrained to 0.91 (2) Å and with *U*_iso_(H) = 1.2*U*_eq_(N).

### Crystal data

**Table:**

C_19_H_13_Cl_2_NO_3_S	*F*_000_ = 832
*M**_r_* = 406.26	*D*_x_ = 1.513 Mg m^−^^3^
Monoclinic, *P*2_1_/*n*	Mo *K*α radiation λ = 0.71073 Å
Hall symbol: -P 2yn	Cell parameters from 31954 reflections
*a* = 8.2307 (1) Å	θ = 0.4–28.3º
*b* = 18.5014 (3) Å	µ = 0.50 mm^−^^1^
*c* = 12.1364 (2) Å	*T* = 173 (2) K
β = 105.211 (1)º	Block, yellow
*V* = 1783.38 (5) Å^3^	0.25 × 0.25 × 0.15 mm
*Z* = 4	

### Data collection

**Table:**

Bruker Kappa APEXII CCD diffractometer	3534 reflections with *I* > 2σ(*I*)
Radiation source: fine-focus sealed tube	*R*_int_ = 0.044
Monochromator: graphite	θ_max_ = 28.3º
*T* = 173(2) K	θ_min_ = 2.1º
φ and ω scans	*h* = −10→10
Absorption correction: none	*k* = −24→24
13987 measured reflections	*l* = −14→16
4401 independent reflections	

### Refinement

**Table:**

Refinement on *F*^2^	Secondary atom site location: difference Fourier map
Least-squares matrix: full	Hydrogen site location: inferred from neighbouring sites
*R*[*F*^2^ > 2σ(*F*^2^)] = 0.042	H atoms treated by a mixture of independent and constrained refinement
*wR*(*F*^2^) = 0.084	*w* = 1/[σ^2^(*F*_o_^2^) + (0.0187*P*)^2^ + 1.5712*P*] where *P* = (*F*_o_^2^ + 2*F*_c_^2^)/3
*S* = 1.05	(Δ/σ)_max_ < 0.001
4401 reflections	Δρ_max_ = 0.32 e Å^−^^3^
238 parameters	Δρ_min_ = −0.31 e Å^−^^3^
1 restraint	Extinction correction: none
Primary atom site location: structure-invariant direct methods	

### Special details

**Table:**

Geometry. All e.s.d.'s (except the e.s.d. in the dihedral angle between two l.s. planes) are estimated using the full covariance matrix. The cell e.s.d.'s are taken into account individually in the estimation of e.s.d.'s in distances, angles and torsion angles; correlations between e.s.d.'s in cell parameters are only used when they are defined by crystal symmetry. An approximate (isotropic) treatment of cell e.s.d.'s is used for estimating e.s.d.'s involving l.s. planes.
Refinement. Refinement of *F*^2^ against ALL reflections. The weighted *R*-factor *wR* and goodness of fit *S* are based on *F*^2^, conventional *R*-factors *R* are based on *F*, with *F* set to zero for negative *F*^2^. The threshold expression of *F*^2^ > σ(*F*^2^) is used only for calculating *R*-factors(gt) *etc*. and is not relevant to the choice of reflections for refinement. *R*-factors based on *F*^2^ are statistically about twice as large as those based on *F*, and *R*- factors based on ALL data will be even larger.

### Fractional atomic coordinates and isotropic or equivalent isotropic displacement parameters (Å^2^)

**Table:**

	*x*	*y*	*z*	*U*_iso_*/*U*_eq_	
Cl1	0.44574 (7)	0.01345 (3)	0.80411 (4)	0.03185 (13)	
S1	0.95358 (6)	0.13899 (3)	0.46317 (4)	0.02381 (11)	
Cl2	0.64457 (7)	0.44781 (3)	0.45821 (5)	0.03514 (13)	
O1	0.47941 (18)	0.13671 (8)	0.33815 (11)	0.0301 (3)	
O3	1.00797 (18)	0.13033 (8)	0.36105 (13)	0.0331 (3)	
O2	1.06708 (17)	0.12706 (8)	0.57308 (12)	0.0310 (3)	
N1	0.7971 (2)	0.08200 (9)	0.44914 (14)	0.0239 (3)	
H1	0.729 (2)	0.0856 (12)	0.3815 (14)	0.029*	
C4	0.7144 (3)	0.01218 (10)	0.71469 (17)	0.0270 (4)	
H4	0.7690	−0.0164	0.7787	0.032*	
C15	0.7816 (2)	0.25730 (11)	0.35920 (17)	0.0261 (4)	
H15	0.7689	0.2312	0.2900	0.031*	
C7	0.4749 (2)	0.15092 (10)	0.43573 (16)	0.0220 (4)	
C6	0.4742 (2)	0.08317 (10)	0.61488 (16)	0.0231 (4)	
H6	0.3652	0.1018	0.6107	0.028*	
C1	0.5564 (2)	0.10128 (10)	0.53194 (16)	0.0215 (4)	
C14	0.8692 (2)	0.22691 (10)	0.46257 (16)	0.0227 (4)	
C10	0.2063 (3)	0.31757 (12)	0.3887 (2)	0.0358 (5)	
H10	0.1250	0.3397	0.3278	0.043*	
C17	0.7328 (2)	0.36241 (10)	0.45946 (17)	0.0246 (4)	
C18	0.8210 (3)	0.33297 (11)	0.56290 (17)	0.0279 (4)	
H18	0.8345	0.3594	0.6318	0.033*	
C16	0.7129 (3)	0.32578 (11)	0.35766 (17)	0.0268 (4)	
H16	0.6530	0.3472	0.2876	0.032*	
C2	0.7158 (2)	0.07170 (10)	0.53854 (16)	0.0220 (4)	
C8	0.3942 (2)	0.21889 (10)	0.46066 (16)	0.0222 (4)	
C19	0.8891 (2)	0.26433 (11)	0.56418 (17)	0.0261 (4)	
H19	0.9491	0.2431	0.6343	0.031*	
C9	0.2776 (3)	0.25261 (11)	0.37109 (17)	0.0280 (4)	
H9	0.2473	0.2307	0.2977	0.034*	
C3	0.7952 (2)	0.02892 (10)	0.63138 (17)	0.0262 (4)	
H3	0.9055	0.0111	0.6376	0.031*	
C5	0.5527 (2)	0.03774 (10)	0.70344 (16)	0.0232 (4)	
C13	0.4391 (3)	0.25198 (11)	0.56727 (17)	0.0266 (4)	
H13	0.5174	0.2291	0.6290	0.032*	
C11	0.2525 (3)	0.35055 (11)	0.4944 (2)	0.0362 (5)	
H11	0.2036	0.3955	0.5058	0.043*	
C12	0.3699 (3)	0.31836 (11)	0.58384 (19)	0.0334 (5)	
H12	0.4030	0.3415	0.6562	0.040*	

### Atomic displacement parameters (Å^2^)

**Table:**

	*U*^11^	*U*^22^	*U*^33^	*U*^12^	*U*^13^	*U*^23^
Cl1	0.0399 (3)	0.0286 (3)	0.0302 (3)	−0.0016 (2)	0.0148 (2)	0.0021 (2)
S1	0.0222 (2)	0.0227 (2)	0.0275 (3)	0.00388 (18)	0.00828 (19)	0.00123 (19)
Cl2	0.0431 (3)	0.0224 (2)	0.0419 (3)	0.0072 (2)	0.0147 (2)	−0.0016 (2)
O1	0.0341 (8)	0.0324 (8)	0.0215 (7)	0.0044 (6)	0.0029 (6)	−0.0029 (6)
O3	0.0374 (8)	0.0304 (8)	0.0375 (8)	0.0066 (6)	0.0202 (7)	0.0006 (6)
O2	0.0221 (7)	0.0336 (8)	0.0349 (8)	0.0041 (6)	0.0031 (6)	0.0044 (6)
N1	0.0255 (8)	0.0219 (8)	0.0239 (9)	−0.0003 (7)	0.0058 (7)	−0.0030 (7)
C4	0.0304 (10)	0.0195 (9)	0.0283 (10)	0.0012 (8)	0.0029 (8)	0.0045 (8)
C15	0.0321 (11)	0.0250 (10)	0.0210 (10)	0.0016 (8)	0.0064 (8)	−0.0016 (8)
C7	0.0191 (9)	0.0223 (9)	0.0229 (10)	−0.0021 (7)	0.0024 (7)	−0.0010 (7)
C6	0.0217 (9)	0.0202 (9)	0.0267 (10)	0.0005 (7)	0.0050 (8)	−0.0016 (8)
C1	0.0227 (9)	0.0169 (9)	0.0223 (9)	−0.0004 (7)	0.0013 (7)	−0.0017 (7)
C14	0.0226 (9)	0.0223 (9)	0.0243 (10)	0.0010 (7)	0.0079 (8)	0.0013 (7)
C10	0.0364 (12)	0.0279 (11)	0.0399 (13)	0.0081 (9)	0.0042 (10)	0.0105 (10)
C17	0.0255 (10)	0.0184 (9)	0.0312 (11)	0.0003 (7)	0.0099 (8)	−0.0014 (8)
C18	0.0327 (11)	0.0268 (10)	0.0242 (10)	−0.0010 (8)	0.0075 (8)	−0.0051 (8)
C16	0.0299 (10)	0.0258 (10)	0.0234 (10)	0.0030 (8)	0.0047 (8)	0.0017 (8)
C2	0.0243 (9)	0.0179 (9)	0.0232 (9)	0.0003 (7)	0.0051 (7)	−0.0014 (7)
C8	0.0201 (9)	0.0205 (9)	0.0255 (10)	−0.0004 (7)	0.0051 (7)	0.0038 (7)
C19	0.0265 (10)	0.0276 (10)	0.0222 (10)	−0.0007 (8)	0.0031 (8)	0.0004 (8)
C9	0.0309 (11)	0.0253 (10)	0.0251 (10)	0.0009 (8)	0.0027 (8)	0.0052 (8)
C3	0.0235 (10)	0.0200 (9)	0.0336 (11)	0.0025 (7)	0.0046 (8)	0.0010 (8)
C5	0.0292 (10)	0.0181 (9)	0.0223 (9)	−0.0034 (8)	0.0067 (8)	−0.0016 (7)
C13	0.0303 (10)	0.0253 (10)	0.0230 (10)	−0.0003 (8)	0.0051 (8)	0.0017 (8)
C11	0.0416 (13)	0.0198 (10)	0.0519 (14)	0.0049 (9)	0.0205 (11)	0.0053 (10)
C12	0.0439 (13)	0.0251 (10)	0.0350 (12)	−0.0047 (9)	0.0168 (10)	−0.0043 (9)

### Geometric parameters (Å, °)

**Table:**

Cl1—C5	1.742 (2)	C14—C19	1.386 (3)
S1—O2	1.4307 (15)	C10—C9	1.379 (3)
S1—O3	1.4329 (15)	C10—C11	1.381 (3)
S1—N1	1.6383 (17)	C10—H10	0.950
S1—C14	1.7679 (19)	C17—C16	1.381 (3)
Cl2—C17	1.7374 (19)	C17—C18	1.386 (3)
O1—C7	1.223 (2)	C18—C19	1.387 (3)
N1—C2	1.429 (2)	C18—H18	0.950
N1—H1	0.865 (15)	C16—H16	0.950
C4—C3	1.384 (3)	C2—C3	1.392 (3)
C4—C5	1.385 (3)	C8—C13	1.391 (3)
C4—H4	0.950	C8—C9	1.394 (3)
C15—C16	1.386 (3)	C19—H19	0.950
C15—C14	1.391 (3)	C9—H9	0.950
C15—H15	0.950	C3—H3	0.950
C7—C8	1.490 (3)	C13—C12	1.391 (3)
C7—C1	1.499 (3)	C13—H13	0.950
C6—C5	1.384 (3)	C11—C12	1.384 (3)
C6—C1	1.393 (3)	C11—H11	0.950
C6—H6	0.950	C12—H12	0.950
C1—C2	1.404 (3)		
			
O2—S1—O3	120.89 (9)	C17—C18—C19	118.95 (18)
O2—S1—N1	107.51 (9)	C17—C18—H18	120.5
O3—S1—N1	104.68 (9)	C19—C18—H18	120.5
O2—S1—C14	107.79 (9)	C17—C16—C15	118.98 (18)
O3—S1—C14	108.11 (9)	C17—C16—H16	120.5
N1—S1—C14	107.13 (9)	C15—C16—H16	120.5
C2—N1—S1	121.25 (13)	C3—C2—C1	120.09 (18)
C2—N1—H1	114.7 (15)	C3—C2—N1	118.44 (17)
S1—N1—H1	110.0 (15)	C1—C2—N1	121.43 (17)
C3—C4—C5	119.05 (18)	C13—C8—C9	119.28 (18)
C3—C4—H4	120.5	C13—C8—C7	122.45 (17)
C5—C4—H4	120.5	C9—C8—C7	118.12 (17)
C16—C15—C14	119.66 (18)	C14—C19—C18	119.64 (18)
C16—C15—H15	120.2	C14—C19—H19	120.2
C14—C15—H15	120.2	C18—C19—H19	120.2
O1—C7—C8	120.44 (17)	C10—C9—C8	120.21 (19)
O1—C7—C1	120.10 (17)	C10—C9—H9	119.9
C8—C7—C1	119.43 (16)	C8—C9—H9	119.9
C5—C6—C1	119.39 (17)	C4—C3—C2	120.33 (18)
C5—C6—H6	120.3	C4—C3—H3	119.8
C1—C6—H6	120.3	C2—C3—H3	119.8
C6—C1—C2	119.26 (17)	C6—C5—C4	121.67 (18)
C6—C1—C7	120.47 (17)	C6—C5—Cl1	118.85 (15)
C2—C1—C7	120.26 (17)	C4—C5—Cl1	119.48 (15)
C19—C14—C15	120.85 (18)	C12—C13—C8	120.28 (19)
C19—C14—S1	120.09 (15)	C12—C13—H13	119.9
C15—C14—S1	119.06 (15)	C8—C13—H13	119.9
C9—C10—C11	120.3 (2)	C10—C11—C12	120.3 (2)
C9—C10—H10	119.9	C10—C11—H11	119.9
C11—C10—H10	119.9	C12—C11—H11	119.9
C16—C17—C18	121.92 (18)	C11—C12—C13	119.7 (2)
C16—C17—Cl2	119.17 (15)	C11—C12—H12	120.2
C18—C17—Cl2	118.92 (15)	C13—C12—H12	120.2
			
O2—S1—N1—C2	46.32 (17)	C7—C1—C2—N1	−6.2 (3)
O3—S1—N1—C2	176.05 (14)	S1—N1—C2—C3	−78.1 (2)
C14—S1—N1—C2	−69.30 (16)	S1—N1—C2—C1	104.42 (19)
C5—C6—C1—C2	1.4 (3)	O1—C7—C8—C13	−154.24 (19)
C5—C6—C1—C7	−179.43 (17)	C1—C7—C8—C13	23.5 (3)
O1—C7—C1—C6	−136.12 (19)	O1—C7—C8—C9	21.4 (3)
C8—C7—C1—C6	46.2 (2)	C1—C7—C8—C9	−160.91 (17)
O1—C7—C1—C2	43.0 (3)	C15—C14—C19—C18	0.0 (3)
C8—C7—C1—C2	−134.72 (18)	S1—C14—C19—C18	−179.36 (15)
C16—C15—C14—C19	−0.2 (3)	C17—C18—C19—C14	0.6 (3)
C16—C15—C14—S1	179.15 (15)	C11—C10—C9—C8	1.5 (3)
O2—S1—C14—C19	−13.39 (18)	C13—C8—C9—C10	−0.8 (3)
O3—S1—C14—C19	−145.63 (16)	C7—C8—C9—C10	−176.61 (19)
N1—S1—C14—C19	102.04 (17)	C5—C4—C3—C2	0.9 (3)
O2—S1—C14—C15	167.28 (15)	C1—C2—C3—C4	3.3 (3)
O3—S1—C14—C15	35.04 (18)	N1—C2—C3—C4	−174.15 (17)
N1—S1—C14—C15	−77.28 (17)	C1—C6—C5—C4	2.9 (3)
C16—C17—C18—C19	−0.9 (3)	C1—C6—C5—Cl1	−178.11 (14)
Cl2—C17—C18—C19	178.95 (15)	C3—C4—C5—C6	−4.0 (3)
C18—C17—C16—C15	0.7 (3)	C3—C4—C5—Cl1	176.93 (15)
Cl2—C17—C16—C15	−179.17 (15)	C9—C8—C13—C12	−0.7 (3)
C14—C15—C16—C17	−0.1 (3)	C7—C8—C13—C12	174.83 (18)
C6—C1—C2—C3	−4.5 (3)	C9—C10—C11—C12	−0.5 (3)
C7—C1—C2—C3	176.37 (17)	C10—C11—C12—C13	−1.1 (3)
C6—C1—C2—N1	172.92 (17)	C8—C13—C12—C11	1.7 (3)

### Hydrogen-bond geometry (Å, °)

**Table:**

*D*—H···*A*	*D*—H	H···*A*	*D*···*A*	*D*—H···*A*
N1—H1···Cl1^i^	0.865 (15)	2.967 (19)	3.6519 (17)	137.5 (18)
N1—H1···O1	0.865 (15)	2.20 (2)	2.798 (2)	126.1 (18)

Symmetry codes: (i) −*x*+1, −*y*, −*z*+1.
